# Prevalence of drug resistant tuberculosis and its associated factors among tuberculosis patients at wolkite health center in central Ethiopia

**DOI:** 10.1038/s41598-026-34986-9

**Published:** 2026-01-08

**Authors:** Asnake Simieneh, Rahel Dereje, Tadesse Misganaw, Gossa Fetene Abebe

**Affiliations:** 1 Armauer Hansen Research Institute (AHRI), Addis Ababa, Ethiopia; 2 School of Public Health, College of Medicine and Health Sciences, Mizan -Tepi University, Mizan-Aman, Ethiopia; 3 Department of Medical Laboratory Sciences, College of Health Sciences, Woldia University, Woldia, Ethiopia; 4 Department of Midwifery, College of Medicine and Health Sciences, Mizan -Tepi University, Mizan-Aman, Ethiopia

**Keywords:** Immunology, Microbiology, Molecular biology, Medical research

## Abstract

Multidrug-resistant tuberculosis (MDR-TB) is increasingly recognized as a global public health challenge. Continuous monitoring of the prevalence of MDR-TB and its associated factors is crucial for the effective control and management of this disease. While alarming rates of MDR-TB have been reported in various parts of Ethiopia, data from rural health settings like Wolkite Health Center remain limited and underexplored. We aim to assess the prevalence of MDR-TB and its associated factors among tuberculosis (TB) patients at Wolkite Health Center. A three-year institution-based retrospective cross-sectional study was conducted at Wolkite Health Center, in the central Ethiopian region, Ethiopia. We have included all tuberculosis patients recorded in the tuberculosis (TB) registration form from February 4, 2021, to December 31, 2023. Data collection was carried out using a pretested structured data extraction checklist. Epidata version 3.1 and SPSS version 22 were used for data entry and analysis, respectively. Binary logistic regression was employed to assess the association between dependent and independent variables. A p-value of less than 0.05 was considered statistically significant. A total of 452 TB cases were included in our study. Of the total TB cases, around 10.4% (47/452) were rifampicin-resistant(RR) TB. Approximately 28.1% (127 out of 452) of the participants reported having been infected with TB at some point in their lifetime. The prevalence of RR TB remains relatively stable, with rates of 10.2% (15 out of 147 cases) in 2021, 8.7% (13 out of 149 cases) in 2022, and 12.2% (19 out of 156 cases) in 2023. Variables such as age, history of previous TB, and HIV status were found to be significantly associated with drug-resistant tuberculosis. Previous TB diseases were 5.1 times [AOR = 5.1, 95% CI: (2.5–10.1)], and HIV-positive TB cases were 6.6 times [AOR = 6.6, 95% CI: (3.5–9.7)] more likely to develop RR TB, whereas young adults aged 18 to 34 have a 95% lower risk of developing rifampicin-resistant tuberculosis compared to older adults over 60 years old [AOR = 0.05, 95% CI: (0.02–0.26)]. As a conclusion, over the three-year period from 2021 to 2023, there is no significant change in the prevalence of RR-TB. The significant association of previous TB treatment, HIV status, and age with drug resistance highlights the need for targeted interventions and comprehensive care strategies.

**Supplementary Information:**

The online version contains supplementary material available at 10.1038/s41598-026-34986-9.

## Introduction

Tuberculosis (TB) remains a significant global health threat, with the 2024 World Health Organization (WHO) reporting approximately 10.8 million infections and 1.25 million deaths attributed to the disease in 2023^1^. Despite the implementation of the End TB Strategy, the global incidence rate is estimated to have increased by 2.2% between 2021 and 2022, highlighting the ongoing challenges in TB management. This situation is exacerbated by the emergence of drug-resistant TB, particularly multidrug-resistant tuberculosis and extensively drug-resistant tuberculosis (XDR-TB), which pose substantial public health challenges^2^. In 2023, around half a million of the total notified TB cases were classified as multidrug-resistant tuberculosis^1^, highlighting the urgent need for enhanced surveillance, innovative treatment approaches, and strengthened public health interventions to address this growing threat.

As of 2024, Ethiopia is among the top 30 countries globally with a high TB burden^1^. The country’s struggle against TB is exacerbated by a high prevalence of various socio-economic factors, including poverty, malnutrition, and an escalating HIV/AIDS epidemic. While significant progress has been made in TB control, evidenced by increased case detection^3^ and treatment success rates^4^, the emergence of drug-resistant strains continues to hinder these achievements. MDR-TB specifically is defined as TB that is resistant to at least isoniazid and rifampicin, the two most effective first-line anti-TB drugs. This resistance can arise from inadequate treatment regimens, poor healthcare infrastructure, and lack of patient adherence to therapy. The rise of MDR-TB not only complicates treatment efficacy but also significantly increases healthcare costs and leads to higher morbidity and mortality rates, delaying the global targets for TB reduction^2,5^. Therefore, there is a pressing need to focus on the challenges posed by multidrug-resistant tuberculosis.

In Ethiopia, the prevalence of MDR-TB is a growing concern, with recent estimates suggesting that about 1.5% of new TB cases and 11% of previously treated cases may be MDR-TB^6^. The WHO global tuberculosis report of 2022 noted that Ethiopia contributes significantly to the global burden of MDR-TB^7^. The emergence of this MDR-TB is largely attributed to several factors, including ineffective treatment practices, health system challenges, and the societal stigma associated with TB that may hinder individuals from seeking timely care. Furthermore, comorbidities such as HIV significantly exacerbate the situation for individuals co-infected with tuberculosis, as studies show that these patients not only experience poorer health outcomes but also face a much higher risk of developing drug-resistant forms of TB^8–10^. Factors such as HIV coinfection further worsen the condition of MDR-TB patients by resulting in high mortality among individuals who have initiated treatment for drug-resistant tuberculosis^11^.

Research conducted in various regions of Ethiopia, including Addis Ababa^8^, Adama^12^, Yirgalem^13^, and Gondar^14^, has revealed alarming rates of resistance and underscores the need for ongoing surveillance and targeted interventions. However, data from rural health settings, such as the Wolkite Health Center, remain limited and underexplored.

This study aims to fill this knowledge gap by analyzing the prevalence of MDR-TB and identifying its associated factors among TB patients at Wolkite Health Center. By providing vital insights into the epidemiology of drug resistance in this context, the findings could inform local and national TB control strategies. Understanding these dynamics is essential for developing tailored interventions that ensure better management of TB patients and ultimately reduce the burden of this disease in Ethiopia. The objective of this study is to determine the prevalence of MDR-TB and its associated factors among TB patients at the Wolkite Health Center in Ethiopia.

## Methods

### Study area and study period

The study was conducted at Wolkite Health Center, Central Ethiopian region, in Ethiopia. Wolkite Health Center is located in Wolkite Town, 158 km southwest of Addis Ababa, the capital city of Ethiopia. The Health Center has an antenatal care (ANC) unit, an outpatient ward, an inpatient ward, an emergency ward, a TB clinic, and a laboratory. Tuberculosis is diagnosed with the GeneXpert assay and registered in the TB laboratory of the health center. The data was collected from March 01 to 31, 2024, on tuberculosis patients who had registered Xpert MTB/RIF TB results between February 04, 2021, and December 31, 2023.

### Study design

This study employs a retrospective cross-sectional design, specifically focusing on a record-based review of tuberculosis registry data.

### Population

#### Source population

The source population included all TB patients who registered at the TB clinic between February 4, 2021, and December 31, 2023.

#### Study population

The study involved retrieving records of all TB cases that had complete information from February 4, 2021, and December 31, 2023.

### Inclusion and exclusion criteria

The study included all TB patients with complete information from February 4, 2021, to December 31, 2023; patients with missing information related to the outcome variable were excluded.

### Sample size and sampling procedure

A total of 1,600 individuals suspected of having TB provided specimens for diagnosis, and 470 cases tested positive using the Xpert MTB/RIF assay between February 4, 2021, and December 31, 2023. Of these, 18 participants were excluded from the study because, although they tested positive for tuberculosis, their resistance status was not recorded, and 452 TB patients met the inclusion criteria and were included in the study.

### Study variables

In this study, the dependent variable was TB positivity, while the independent variables included age, gender, residence, HIV status, type of TB, patient type, and history of previous TB infection (S1 file).

### Data collection period and data quality

Data for this research was collected from the TB register of Wolkite Health Center using a standardized checklist by the data collectors from **March 1 to March 31**,** 2024**. The checklist included socio-demographic characteristics, HIV status, types and categories of TB, and the history of previous TB infections. To ensure data quality, two laboratory professionals collected the data after receiving one day of training on the data collection format and techniques. The data was checked for its completeness every day by the principal investigator.

### Data management and analysis

The data were entered and cleaned using EpiData version 3.1 and then exported to SPSS version 25 for analysis. Descriptive statistics, including frequencies and percentages, were computed. Bivariate and multivariable logistic regression analyses were conducted to examine the associations between the outcome variable and independent variables. During the bivariate analysis, variables with a p-value less than 0.25 were selected for inclusion in the multivariable logistic regression. The Hosmer-Lemeshow goodness-of-fit test was performed to assess model fit (p-value = 0.64), and multicollinearity was evaluated, with all independent variables showing no collinearity (VIF < 10). Variables with a p-value less than 0.05 were considered statistically significant and reported with odds ratios (OR) and 95% confidence intervals (CI).

### Operational definitions

#### Pulmonary TB (PTB)

Tuberculosis that primarily affects the lung.

**Extra-pulmonary TB (EPTB**): Tuberculosis that affects organs other than the lungs, such as the lymph nodes, abdomen, genitourinary system, skin, joints, bones, and the meninges.

#### New TB case

New TB cases are individuals who have been diagnosed with tuberculosis for the first time and have never received any prior treatment for the disease.

#### Previous history of TB

individuals who have been diagnosed with active TB in the past and have either completed treatment or were treated unsuccessfully^15^.

### Ethical consideration

Before we started the study, ethical approval was obtained from the Institutional Review Board of Wolkite University College of Medicine and Health Sciences (Ref No. WKU IRB 405/2024). Permission was obtained from the Health Center administration before data collection. This research was conducted retrospectively and did not involve direct interaction with individuals. In accordance with the ethical guidelines established by the Institutional Review Board of Wolkite University College of Medicine and Health Sciences, informed consent was waived for this study. However, the information obtained was made anonymous and de-identified prior to analysis to ensure confidentiality. All methods were performed in accordance with the relevant guidelines and regulations.

## Results

### Sociodemographic and clinical characteristics

A total of 452 cases were included in our study. Of the total TB cases, around 10.4% (47/452) were rifampicin-resistant TB. The majority of our participants are males, with 64.2% (290/452). Regarding the age groups, the majority of participants were aged 18–34, comprising 39.4% (178/452) of individuals, followed by those aged 35–49, who accounted for 29.6% (134/452) of cases. In terms of residence, most participants lived in rural areas, with 53.5% (242/452) individuals compared to people from urban settings. In terms of HIV status, about 23.6% (107/452) of the TB cases are HIV positive. Regarding the patient types, the majority were new cases, totaling 71.9% (325/452), followed by 25.4% (115/452) relapsed cases. In this study the majority of the TB cases were pulmonary TB cases, 71% (321/452). Regarding prior TB infection, approximately 28.1% (127 out of 452) of the participants reported having been infected with TB at some point in their lifetime (Table 1).

**Table 1:** Sociodemographic and clinical characteristics of RR TB cases at wolkite health Center, Ethiopia, from February 04, 2021, to December 31, 2023.

Variables	Frequency (%)	RR TB status	
		Positive (%)	Negative (%)
Gender			
Male	290(64.2)	34(11.7)	256(88.3)
Female	162(35.8)	13(8)	149(92)
Age			
18–34	178(39.4)	12(6.7)	166(93.3)
35–49	134(29.6)	14(10.4)	120(89.6)
50–64	103(22.8)	15(14.5)	88(85.5)
> 65	37(8.2)	6(16.2)	31(83.8)
Residence			
Urban	210(46.5)	24(11.4)	186(88.6)
Rural	242(53.5)	23(9.5)	219(90.5)
History of previous TB			
Yes	127(28.1)	31(24.4)	96(75.6)
No	325(71.9)	16(5)	309(95)
HIV status			
Positive	107(23.6)	23(21.5)	84(78.5)
Negative	345(76.4)	24(6.9)	321(93.1)
Patient types			
New cases	325(71.9)	16(5)	309(95)
Relapsed	115(25.4)	29(25.2)	86(74.8)
Treatment failure	5(1.1)	0	5(100%)
After lost follow-up	7(1.5)	2(28.6)	5(71.4)
Type of TB			
PTB	321 (71)	34(10.6)	287(89.4)
EPTB	131 (29)	13(9.9)	118(90.1)
Total	452	47(10.4)	405(89.6)

### Prevalence of RR tuberculosis

**Fig. 1:**
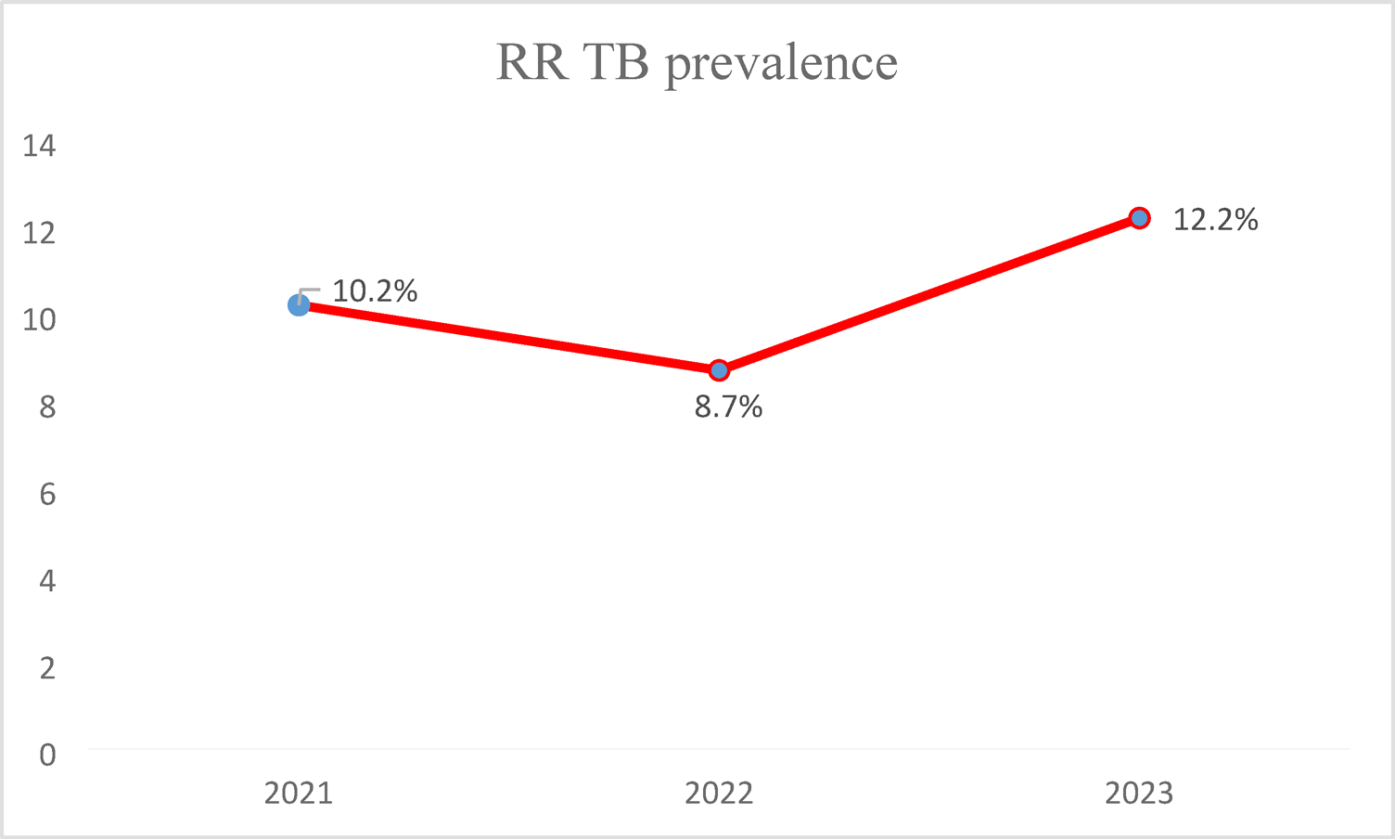
The trend of RR-TB among TB-confirmed patients at Wolkite Health Center, Central Ethiopia Regional State, in Ethiopia from February 04, 2021, to December 31, 2023.

Out of the total TB cases analyzed, 47 were identified as RR-TB, resulting in an overall prevalence of 10.4%. The prevalence of RR TB among previously TB-treated cases was 24.4% (31/127), whereas the prevalence among new TB cases was only 4.9% (16/325). The overall prevalence of RR TB varied slightly each year, with rates of 10.2% (15 out of 147 cases) in 2021, 8.7% (13 out of 149 cases) in 2022, and 12.2% (19 out of 156 cases) in 2023 (Fig. 1). The chi-square test was conducted to determine whether there was a significant difference in the prevalence of RR-TB over the three-year period. The results showed a chi-square statistic of x^2^ = 0.985 and a p-value of 0.611. These findings indicate that there is no significant difference in the prevalence of RR-TB among the years 2021, 2022, and 2023.

### Factors associated with RR TB cases at wolkite health center

We conducted a logistic regression analysis to see the association of sociodemographic and clinical factors with drug-resistant tuberculosis. In the bivariate logistic regression analysis, participant’s sex, age, previous history of tuberculosis, and HIV status were independent variables found to have an association with drug-resistant TB. However, after the confounding variables were adjusted in multivariable logistic regression, except for sex, the other variables, such as age, history of previous TB, and HIV status, were found to be significantly associated with drug-resistant tuberculosis.

In our study, those participants who had a history of previous TB diseases were 5.1 times [AOR = 5.1, 95% CI: (2.5–10.1)] more likely to develop anti-TB drug resistance than those who don’t have a history of previous TB (new TB cases).

Participants who were HIV positive were 6.6 times more likely to develop anti-TB drug resistance than those participants who were HIV negative TB cases [AOR = 6.6, 95% CI: (3.5–9.7)]. Compared to the elderly TB cases, young adult TB cases have a 95% lower risk of developing anti-TB drug resistance [AOR = 0.05, 95% CI: (0.02–0.26)] (Table 2).

**Table 2:** Bivariate and multivariable logistic regression analysis of factors associated with RR TB cases at wolkite health Center, from February 04, 2021, to December 31, 2023.

Variables			Frequency	RR TB status		COR(95%CI)		*p*-value		AOR(95%CI)		*p*-value
				Positive	Negative							
Sex												
Male			290	34	256	1.5 (0.787-2.9)		0.22		1.6 (0.8–3.4)		0.2
Female			162	13	149	Ref		Ref		Ref		Ref
Age												
18–34			178	12	166	0.37 (0.13–1.1)		0.06		0.05 (0.02–0.26)		< 0.001
35–49			134	14	120	0.6 (0.2–1.7)		0.33		0.23 (0.06–0.85)		0.027
50–64			103	15	88	0.9 (0.3–2.5)		0.8		0.84 (0.28–2.5)		0.75
> 65			37	6	31	Ref		Ref		Ref		Ref
Residence												
Urban			210	24	186	Ref				-		-
Rural			242	23	219	0.8 (0.4–1.5)		0.5		-		-
History of previous TB												
Yes			127	31	96	6.2 (3.3–11.9)		< 0.001		5.1(2.5–10.1)		< 0.001
No			325	16	309	Ref		Ref		Ref		Ref
HIV												
Positive			107	23	84	3.6 (1.9–6.8)		< 0.001		6.6 (3.5–9.7)		< 0.001
Negative			345	24	321	Ref		Ref		Ref		Ref
Type of TB												
PTB			321	34	287	1.1 (0.5–2.1)		0.8		-		-
EPTB			131	13	118	Ref		Ref		-		-

## Discussion

This study aimed to determine the prevalence of RR-TB and its associated factors among TB patients at Wolkite Health Center over a three-year period from 2021 to 2023.

In our study, the overall prevalence of RR TB among confirmed tuberculosis cases is 10.4% (95% CI: 7.2% to 13.5%). This finding aligns with previous research conducted in Adama^12^ and corresponds with the national prevalence of RR TB reported in literature^16^. This result highlights the potential shortcomings in the effectiveness and implementation of World Health Organization (WHO) guidelines designed to combat drug-resistant TB in Ethiopia^17^. Given these concerning results, there is a pressing need for further research to explore the underlying factors contributing to this high rate of RR TB. Identifying these determinants is crucial for developing effective strategies to mitigate the spread of drug-resistant strains and improve TB management practices within the country.

In this study the prevalence of RR-TB among previously treated TB cases was notably higher at 24.4% compared to just 4.9% among new TB cases. This finding underscores the critical importance of monitoring and managing treatment regimens for individuals with a history of TB, as they are at a greater risk of developing drug resistance. This is because they may not have taken their TB medication properly, or they may have experienced a relapse^18^.

In our study over the three-year period from 2021 to 2023, we observed no significant change in the prevalence of RR-TB. This finding aligns with the World Health Organization report, which indicates that the number of individuals developing MDR/RR-TB has remained relatively stable from 2020 to 2023, as noted in their 2024 report^1^. This situation highlights the need for more targeted public health interventions and increased investment in TB control strategies, especially in high-burden areas. Continued monitoring, research, and adaptable health strategies are crucial to addressing the persistent challenges posed by this public health threat.

Our logistic regression analysis identified several significant factors associated with MDR-TB. Participant’s age, history of previous TB and HIV status are found to be a significant predictor of RR TB.

Regarding the history of previous TB treatment, notably, a history of previous TB treatment was a strong predictor, with individuals having a 5.1 times higher likelihood of developing drug resistance compared to those without such a history. This finding aligns with existing literature^13,14,19–22^, that emphasizes the risks associated with inadequate treatment and the importance of ensuring complete and effective therapy for all TB patients. A previous history of tuberculosis is associated with an increased risk of multidrug-resistant TB primarily due to the potential for incomplete treatment or loss of adherence during earlier TB infections, which can lead to the development of drug resistance^19^.

In this study HIV co-infection emerged as another predictor, with HIV-positive individuals being 6.6 times more likely to develop RR-TB. This highlights the intersection of TB and HIV as a significant public health challenge, particularly in regions where both diseases are prevalent. The immunocompromised state of HIV-positive patients can lead to poorer treatment outcomes and increased susceptibility to drug-resistant strains, necessitating integrated care approaches that address both infections simultaneously. Our finding is in line with previous studies^9,23,24^. Moreover, a systematic review has highlighted that the anti-TB treatment success rate is significantly reduced by HIV co-infection^25^. This is because HIV coinfection leads to malabsorption of anti-TB drugs, such as rifampicin and ethambutol, leading to drug resistance and treatment failure^26^.

In this study, we found that young adults aged 18 to 34 have a 95% lower risk of developing rifampicin-resistant tuberculosis compared to older adults over 60 years old. This significant difference suggests that younger individuals may be less likely to develop MDR-TB due to variations in exposure, health-seeking behaviors, or treatment adherence across different age groups. A study in Greece^27^ reported that geriatric patients are more susceptible to MDR organisms, which may be attributed to the weakening of the immune system as people age, making them more vulnerable to infections. Consequently, this finding implies that targeted interventions may be necessary for older populations, who often face higher risks due to comorbidities or previous treatment failures. Studies in MDR-TB^28,29^ support our results; however, some studies^13,30,31^ indicate that MDR-TB can be more prevalent among younger individuals rather than the elderly, highlighting inconsistencies in existing research. Therefore, comprehensive studies are essential to better understand the impact of age on MDR-TB susceptibility.

### Implications for public health

The results of this study underscore the urgent need for enhanced TB control strategies in Central Ethiopia, particularly focusing on the prevention and management of MDR-TB. Strengthening the capacity for early diagnosis, ensuring adherence to treatment protocols, and providing comprehensive care for co-infected individuals are critical steps in mitigating the impact of drug-resistant TB. Additionally, public health campaigns aimed at raising awareness about the risks associated with previous TB treatment and the importance of regular screening for high-risk populations could play a vital role in reducing the prevalence of MDR-TB.

### Limitation and strength

The results of this study are both timely and significant, particularly in light of the ongoing public health challenges posed by tuberculosis and its drug-resistant forms. The comprehensive data collection over three years enhances the reliability of the findings and provides a solid foundation for public health recommendations. However, the retrospective design may introduce biases and limit the ability to draw causal conclusions. Although the culture test is still considered the gold standard for TB diagnosis, it was not available at Wolkite Health Center during the study period. Consequently, we relied solely on the Xpert MTB/RIF assay for diagnosis. This may affect the comprehensiveness of our findings. Additionally, our study is limited to patients who sought care at Wolkite Health Center, which may impact the generalizability of the findings.

## Conclusions

In conclusion, this study provides valuable insights into the prevalence of RR-TB and its associated factors among TB patients at Wolkite Health Center. The significant association of previous TB treatment, HIV status, and age with drug resistance highlights the need for targeted interventions and comprehensive care strategies. Continued research and surveillance are essential to inform public health policies and improve outcomes for TB patients in the region.

## Supplementary Information

Below is the link to the electronic supplementary material.

Supplementary Material 1

Supplementary Material 2

## Data Availability

All relevant data are available within the paper (S1 file).
