# Supplementary material for: Prevalence of drug resistant tuberculosis and its associated factors among tuberculosis patients at wolkite health center in central Ethiopia
Source: Sci Rep. 2026 Jan 8;16:4888. doi: 10.1038/s41598-026-34986-9 (PMC12873313; doi:10.1038/s41598-026-34986-9)
Supplement: Supplementary file 2 — Supplementary Material 2 [file 41598_2026_34986_MOESM2_ESM.docx]

S1 File: The original SPSS dataset used and analyzed in our study
